# Pathological changes in the lungs and lymphatic organs of twelve COVID-19 autopsy cases

**DOI:** 10.1093/nsr/nwaa247

**Published:** 2020-09-29

**Authors:** Qian Liu, Yu Shi, Jun Cai, Yaqi Duan, Rongshuai Wang, Hongyan Zhang, Qiurong Ruan, Jiansha Li, Lei Zhao, Yifang Ping, Rong Chen, Liang Ren, Xiaochun Fei, Heng Zhang, Rui Tang, Xi Wang, Tao Luo, Xindong Liu, Xuequan Huang, Zhenhua Liu, Qilin Ao, Yong Ren, Jing Xiong, Zhicheng He, Haibo Wu, Wenjuan Fu, Pengnan Zhao, Xinwei Chen, Guoqiang Qu, Yunyun Wang, Xi Wang, Jia Liu, Dongfang Xiang, Sanpeng Xu, Xiaowei Zhou, Qingrui Li, Jinghong Ma, Heng Li, Jie Zhang, Sizhe Huang, Xiaohong Yao, Yiwu Zhou, Chaofu Wang, Dingyu Zhang, Guoping Wang, Liang Liu, Xiu-Wu Bian

**Affiliations:** Department of Forensic Medicine, Tongji Medical College, Huazhong University of Science and Technology, Wuhan, Hubei 430030, China; Institute of Pathology, Southwest Hospital, Third Military Medical University (Army Medical University), Chongqing 400038, China; Key Laboratory of Tumor Immunopathology, Ministry of Education of China, Chongqing 400038, China; Department of Pathology, Shanghai Jiaotong University School of Medicine, Shanghai 200025, China; Institute of Pathology, Tongji Hospital, Tongji Medical College, Huazhong University of Science & Technology, Wuhan, Hubei 430030, China; Department of Pathology, Tongji Medical College, Huazhong University of Science & Technology, Wuhan, Hubei 430030, China; Hubei Chongxin Judicial Expertise Center, Wuhan, Hubei 430415, China; Southwest Hospital, Third Military Medical University (Army Medical University), Chongqing 400038, China; Institute of Pathology, Tongji Hospital, Tongji Medical College, Huazhong University of Science & Technology, Wuhan, Hubei 430030, China; Department of Pathology, Tongji Medical College, Huazhong University of Science & Technology, Wuhan, Hubei 430030, China; Institute of Pathology, Tongji Hospital, Tongji Medical College, Huazhong University of Science & Technology, Wuhan, Hubei 430030, China; Department of Pathology, Tongji Medical College, Huazhong University of Science & Technology, Wuhan, Hubei 430030, China; Department of Pathology, Shanghai Jiaotong University School of Medicine, Shanghai 200025, China; Institute of Pathology, Southwest Hospital, Third Military Medical University (Army Medical University), Chongqing 400038, China; Key Laboratory of Tumor Immunopathology, Ministry of Education of China, Chongqing 400038, China; Department of Pathology, Wuhan Jinyintan Hospital, Wuhan, Hubei 430023, China; Department of Forensic Medicine, Tongji Medical College, Huazhong University of Science and Technology, Wuhan, Hubei 430030, China; Department of Pathology, Ruijin Hospital, Shanghai Jiaotong University School of Medicine, Shanghai 200025, China; Department of Pathology, Ruijin Hospital, Shanghai Jiaotong University School of Medicine, Shanghai 200025, China; Institute of Pathology, Southwest Hospital, Third Military Medical University (Army Medical University), Chongqing 400038, China; Key Laboratory of Tumor Immunopathology, Ministry of Education of China, Chongqing 400038, China; Institute of Pathology, Tongji Hospital, Tongji Medical College, Huazhong University of Science & Technology, Wuhan, Hubei 430030, China; Department of Pathology, Tongji Medical College, Huazhong University of Science & Technology, Wuhan, Hubei 430030, China; Institute of Pathology, Southwest Hospital, Third Military Medical University (Army Medical University), Chongqing 400038, China; Key Laboratory of Tumor Immunopathology, Ministry of Education of China, Chongqing 400038, China; Institute of Pathology, Southwest Hospital, Third Military Medical University (Army Medical University), Chongqing 400038, China; Key Laboratory of Tumor Immunopathology, Ministry of Education of China, Chongqing 400038, China; Department of Vascular Surgery, Southwest Hospital, Third Military Medical University (Army Medical University), Chongqing 400038, China; Department of Pathology, Ruijin Hospital, Shanghai Jiaotong University School of Medicine, Shanghai 200025, China; Institute of Pathology, Tongji Hospital, Tongji Medical College, Huazhong University of Science & Technology, Wuhan, Hubei 430030, China; Department of Pathology, Tongji Medical College, Huazhong University of Science & Technology, Wuhan, Hubei 430030, China; Department of Pathology, General Hospital of Central Theater Command of PLA, Wuhan, Hubei 430070, China; Institute of Pathology, Tongji Hospital, Tongji Medical College, Huazhong University of Science & Technology, Wuhan, Hubei 430030, China; Institute of Pathology, Southwest Hospital, Third Military Medical University (Army Medical University), Chongqing 400038, China; Key Laboratory of Tumor Immunopathology, Ministry of Education of China, Chongqing 400038, China; Department of Pathology, the First Affiliated Hospital of USTC, Division of Life Sciences and Medicine, University of Science and Technology of China, Hefei, Anhui 230036, China; Institute of Pathology, Southwest Hospital, Third Military Medical University (Army Medical University), Chongqing 400038, China; Key Laboratory of Tumor Immunopathology, Ministry of Education of China, Chongqing 400038, China; Southwest Hospital, Third Military Medical University (Army Medical University), Chongqing 400038, China; Department of Pathology, General Hospital of Central Theater Command of PLA, Wuhan, Hubei 430070, China; Hubei Chongxin Judicial Expertise Center, Wuhan, Hubei 430415, China; Department of Forensic Medicine, Tongji Medical College, Huazhong University of Science and Technology, Wuhan, Hubei 430030, China; State Key Laboratory of Virology, Wuhan Institute of Virology, Center for Biosafety Mega-Science, Chinese Academy of Sciences, Wuhan, Hubei 430071, China; State Key Laboratory of Virology, Wuhan Institute of Virology, Center for Biosafety Mega-Science, Chinese Academy of Sciences, Wuhan, Hubei 430071, China; Institute of Pathology, Southwest Hospital, Third Military Medical University (Army Medical University), Chongqing 400038, China; Key Laboratory of Tumor Immunopathology, Ministry of Education of China, Chongqing 400038, China; Institute of Pathology, Tongji Hospital, Tongji Medical College, Huazhong University of Science & Technology, Wuhan, Hubei 430030, China; Hubei Chongxin Judicial Expertise Center, Wuhan, Hubei 430415, China; Institute of Pathology, Southwest Hospital, Third Military Medical University (Army Medical University), Chongqing 400038, China; Key Laboratory of Tumor Immunopathology, Ministry of Education of China, Chongqing 400038, China; Hubei Chongxin Judicial Expertise Center, Wuhan, Hubei 430415, China; Department of Pathology, the First Affiliated Hospital of USTC, Division of Life Sciences and Medicine, University of Science and Technology of China, Hefei, Anhui 230036, China; Department of Forensic Medicine, Tongji Medical College, Huazhong University of Science and Technology, Wuhan, Hubei 430030, China; Department of Forensic Medicine, Tongji Medical College, Huazhong University of Science and Technology, Wuhan, Hubei 430030, China; Institute of Pathology, Southwest Hospital, Third Military Medical University (Army Medical University), Chongqing 400038, China; Key Laboratory of Tumor Immunopathology, Ministry of Education of China, Chongqing 400038, China; Department of Forensic Medicine, Tongji Medical College, Huazhong University of Science and Technology, Wuhan, Hubei 430030, China; Department of Pathology, Ruijin Hospital, Shanghai Jiaotong University School of Medicine, Shanghai 200025, China; Research Center for Translational Medicine, Wuhan Jinyintan Hospital, Wuhan, Hubei 430023, China; Joint Laboratory of Infectious Diseases and Health, Wuhan Institute of Virology and Wuhan Jinyintan Hospital, Chinese Academy of Sciences, Wuhan, Hubei 430023, China; Institute of Pathology, Tongji Hospital, Tongji Medical College, Huazhong University of Science & Technology, Wuhan, Hubei 430030, China; Department of Pathology, Tongji Medical College, Huazhong University of Science & Technology, Wuhan, Hubei 430030, China; Department of Forensic Medicine, Tongji Medical College, Huazhong University of Science and Technology, Wuhan, Hubei 430030, China; Institute of Pathology, Southwest Hospital, Third Military Medical University (Army Medical University), Chongqing 400038, China; Key Laboratory of Tumor Immunopathology, Ministry of Education of China, Chongqing 400038, China

**Keywords:** COVID-19, SARS-CoV-2, Autopsy, Histopathology, Immune disorder

## Abstract

Systematic autopsy and comprehensive pathological analyses of COVID-19 decedents should provide insights into the disease characteristics and facilitate the development of novel therapeutics. In this study, we report the autopsy findings from the lungs and lymphatic organs of twelve COVID-19 decedents that evaluated histopathological changes, immune cell signature, and inflammatory factor expression in the lungs, spleen, and lymph nodes. Here we show that the major pulmonary alternations included diffuse alveolar damage, interstitial fibrosis, and exudative inflammation featured with extensive serous and fibrin exudates, macrophage infiltration, and abundant production of inflammatory factors (IL-6, IP-10, TNFα and IL-1β). The spleen and hilar lymph nodes contained lesions with tissue structure disruption and immune cell dysregulation, including lymphopenia and macrophage accumulation. These findings provide pathological evidence that links injuries of the lungs and lymphatic organs with the fatal systematic respiratory and immune malfunction in critically ill COVID-19 patients.

